# Digital phenotyping of generalized anxiety disorder: using artificial intelligence to accurately predict symptom severity using wearable sensors in daily life

**DOI:** 10.1038/s41398-022-02038-1

**Published:** 2022-08-17

**Authors:** Nicholas C. Jacobson, Brandon Feng

**Affiliations:** 1 Center for Technology and Behavioral Health, Geisel School of Medicine, Dartmouth College, Lebanon, PA USA; 2 Department of Biomedical Data Science, Geisel School of Medicine, Dartmouth College, Lebanon, PA USA; 3 Department of Psychiatry, Geisel School of Medicine, Dartmouth College, Lebanon, PA USA

**Keywords:** Diagnostic markers, Depression

## Abstract

**Background:**

Generalized anxiety disorder (GAD) is a highly prevalent condition. Monitoring GAD symptoms requires substantial time, effort, and cost. The development of digital phenotypes of GAD may enable new scalable, timely, and inexpensive assessments of GAD symptoms.

**Method:**

The current study used passive movement data collected within a large national cohort (*N* = 264) to assess GAD symptom severity.

**Results:**

Using one week of movement data, machine learning models accurately predicted GAD symptoms across a continuum (*r* = 0.511) and accurately detected those individuals with elevated GAD symptoms (AUC = 0.892, 70.0% Sensitivity, 95.5% Specificity, Brier Score = 0.092). Those with a risk score at the 90^th^ percentile or above had 21 times the odds of having elevated GAD symptoms compared to those with lower risk scores. The risk score was most strongly associated with irritability, worry controllability, and restlessness (individual *r*s > 0.5). The risk scores for GAD were also discriminant of major depressive disorder symptom severity (*r* = 0.190).

**Limitations:**

The current study examined the detection of GAD symptom severity rather than the prediction of GAD symptom severity across time. Furthermore, the instant sample of data did not include nighttime actigraphy, as participants were not asked to wear the actigraphs at night.

**Conclusions:**

These results suggest that artificial intelligence can effectively utilize wearable movement data collected in daily life to accurately infer risk of GAD symptoms.

In the United States, Generalized Anxiety Disorder (GAD) is estimated to occur in 1.6% to 5.0% of the general population at some point in their lifetimes [1–3]. GAD is characterized by chronic and uncontrollable worry accompanied by somatic anxiety symptoms [4, 5]. It can be debilitating with many GAD patients experiencing role impairments such as divorce and separation, higher rates of unemployment, and self-reported interference with daily activities [3, 6, 7]. Moreover, GAD also leads to an increased risk of suicidal ideation [8]. GAD also has broad societal implications, as it leads to decreased work productivity and increased healthcare costs [9–14].

Unfortunately, monitoring and assessing GAD symptoms is difficult. Many of these symptoms are naturally hard for patients to recognize or quantify, particularly psychological ones, such as trouble controlling excess worries or general irritability. [15–18] Proper recognition of these symptoms often requires professional consultation through avenues such as structured clinical interviews [5, 19–21], which may be cost and time prohibitive.

Screening tools based on patient self-reporting (such as the GAD-7 and the ASQ-15) are another common instrument. Nevertheless, self-assessment tools for anxiety disorders such as GAD generally show less favorable psychometric characteristics than screens used for depressive disorders. While some, like the GAD-7, have proven relatively reliable, the sheer variety in screening tools and their scoring methods make communication and interpretation difficult [22].

Digital phenotyping -- the use of personal digital devices to provide moment-by-moment quantifications of a person’s daily life - serves as a possible solution to this problem of symptom monitoring and recognition [23]. Wearables and smartphones can collect abundant, varied patient data that can capture information more accurate and relevant to patients’ daily lives than periodic clinical assessments by collecting data with greater ecological validity. If coupled with machine learning, digital phenotyping can help predict symptom dynamics, as well as possibly establish new phenotypes to make future symptom assessments easier and all in a much more cost-effective and convenient way than psychologist visits and other current assessment methods [24].

However, current research in using digital phenotyping to monitor and predict GAD symptoms remains insufficient. First, few studies focus on GAD. One study developed a platform for modeling and analyzing smartphone medical data, even collecting GAD symptoms using mobile formats in an early sample, but focused more on its biostatistical tools than methods for GAD symptom prediction [24]. Although some studies have examined the ability to predict general stress using mobile sensing data [25–30], these may have limited relevance to predicting specific anxiety disorder diagnoses. Likewise, despite their frequent co-occurrence, many studies have examined the utility of digital phenotyping efforts to study depression [31–33], but neglected studying GAD symptoms. That being said, progress has been made in utilizing digital phenotyping to address specific anxiety disorders. Digital phenotyping using smartphone sensor data was able to accurately predict social anxiety disorder symptom severity (*r* = 0.702) [34], with the most important feature being persons’ movement patterns, rather than social features such as calls or texts. Similarly, another study was able to accurately detect pain and worry severity in HIV patients using one week of patient wearable movement data [35]. Although not addressing GAD specifically, these studies demonstrate the potential of using passively collected movement data to predict psychopathology.

Although studies have highlighted the potential theoretical potential of predicting anxiety psychopathology using digital phenotyping [24], only one study has researched the use of passively collected data in daily life to predict GAD symptoms [36]. The study did find a small negative correlation between GAD and time spent at semantic locations. However, these analyses were not performed out-of-sample [36], meaning that the that there was cross-validation of the models (i.e. which could cause problems if the models are overfit). Overall, despite the rapidly increasing use of digital phenotyping in predicting other psychiatric disorders, much more research is needed to examine the potential of utilizing digital phenotyping to predict GAD symptoms. Moreover, in order to maximize the potential clinical utility of digital phenotyping, research should explore the utilization of machine learning to predict GAD and evaluate the predictions based on out-of-sample predictions (i.e. cross-validation).

These models should be used not only for predicting GAD symptom severity, but also for detecting elevated -- or above normal -- symptom severity. Given its poor diagnosis rates [37, 38], detecting individuals at risk for having elevated GAD symptom severity is particularly important, as it can predict the onset of later GAD diagnoses. For example, a study performed on child GAD patients found that symptoms, such as the number of worries and the severity of worries, were good predictors of GAD diagnosis and impairment [39]. Another study performed to assess GAD’s DSM-V symptom criteria found that elevated GAD symptoms and process variables sufficiently differentiated GAD patients from non-anxious controls and patients with different disorders [40].

In addition, it is important to examine the necessity of predicting the totality of symptom risks, as well as the constellation of individual symptoms. In particular, individual GAD symptoms may be a largely unexamined source of clinically relevant data [41–43]. Individual GAD symptoms are also differentially associated with clinical significance to daily life, including social, occupational, and functional ability [44]. Moreover, genetic diatheses have suggested that different genetic profiles may exist for different constellations of GAD symptoms [45]. Thus, this work suggests it is important to examine not only GAD symptoms as a whole, but also to assess the ability to differentially predict different symptoms based on the same risk score.

In the current study, we aimed to test the viability of using passive wearable sensor data, gathered over the course of a week, to predict GAD symptom severity within a large national cohort. The current study used data available from the National Health and Nutrition Examination Study (NHANES) collecting wearable actigraphy data (i.e. capturing daytime movements for one week) [46]. Wearable movement data may have particular relevance to the study of GAD, given that GAD is characterized by psychomotor agitation (e.g., restlessness that results in movements without explicit intent) [47]. Relatedly, movement sensors can also index exercise, which has been shown to have immediate impacts on GAD symptoms [48]. Given this, patterns in daily movement captured from wearable movement data may be highly influential in inferring GAD symptoms. Based on prior related studies, we hypothesized:

**Hypothesis 1:** One week of movement data could accurately predict GAD symptom severity across a continuum.

**Hypothesis 2:** High risk scores developed from machine learning models using the movement data could be used to accurately differentiate those with elevated GAD symptoms (greater than 1 standard deviation above the sample mean on GAD symptom measures) from those with normative GAD symptoms (less than 1 standard deviation above the mean on GAD symptom measures), with an area under the receiver operator curve (AUC) of greater than 0.7.

**Hypothesis 3:** High risk scores from the movement data would be associated with an increase in the risk of having elevated GAD symptoms.

**Hypothesis 4:** Movement risk scores would be most strongly related to somatic GAD symptoms, including trouble sleeping, restlessness, and feeling keyed up, compared with more cognitive GAD symptoms such as the number of different worries, controllability of different worries, and difficulty of getting worries out of one’s mind.

## Method

### Participants

Participants were recruited as part of the National Health and Nutrition Examination Study (NHANES) from 2003–2004. A total of 593 participants (*N* = 593, 55.1% Female, *M*_age_ = 28.95, Age range 20–39, 49.6% Non-Hispanic White, 20.9% Non-Hispanic Black, 20.6% Mexican American, 4.7% Other Hispanic, 4.2% Other Race/Multi-Racial) completed both the generalized anxiety disorder symptom interview as well as the actigraphy study. Based on the weartime algorithms published alongside the NHANES actigraphy data [49, 50], (There was an average of compliance of 33.57% among the non-compliant participants. Note that the correlation between compliance levels and GAD symptom severity was small and non-significant (*r* = −0.06, *p* = .123)) 264 participants actually wore the actigraph for at least half the week (48.5% Female, *M*_age_ = 29.76, Age range 20–39, 53.0% Non-Hispanic White, 21.2% Non-Hispanic Black, 19.7% Mexican American, 3.0% Other Hispanic, 3.0% Other Race/Multi-Racial), thus constituting the sample for the current study.

### Measures

#### Composite international diagnostic interview (CIDI, version 2.1)

The CIDI was used to assess Diagnostic and Statistical Manual (DSM) 5 generalized anxiety disorder symptoms. (Note that although the generalized anxiety disorder was designed around DSM-IV symptoms, the symptoms of GAD have remained equivalent between DSM-IV to DSM-5.) The current measure of Generalized Anxiety Disorder symptom severity was based on questions presented in Table 1. Each of these items were standardized and then summed to create a GAD symptom severity score composite score. The GAD symptom criteria have shown to have high test-retest reliability (retest kappa = 0.69, interrater kappa = 0.96) [51]. Note that a composite measure was also formed for major depressive disorder (MDD) to examine the degree of differentiation of model predictions.

**Table 1:** CIDI Interview Questions to Assess Generalized Anxiety Disorder.

#	Question	Abbreviation
1	“In the past 12 months, did you have a period of a month or more when most days you felt worried or tense or anxious about everyday problems such as work or family?”	Anx > 1 Mo
2	“Did that period go on for at least six months?”	Anx > 6Mo
3	“How many months out of the last 12 did you feel worried or tense or anxious most days?”	Anx Dur
4	“During (that/those) month(s), were you worried, tense, or anxious every day, nearly every day, most days, about half the days, or less than half the days?”	Anx Freq
5	“And on the days you worried or were tense or anxious, did you usually feel that way all day long, most of the day, about half the day, or less than half the day?”	Anx Hours
6	For the past 12 months, “how many months out of the last 12 did you feel worried or tense or anxious most days” were you more worried or tense or anxious compared to most people would be in your same situation?*	GAD Dur
7	“During (that/those) month(s), were you worried, tense, or anxious every day, nearly every day, most days, about half the days, or less than half the days?”	GAD Freq
8	“And on the days you worried or were tense or anxious, did you usually feel that way all day long, most of the day, about half the day, or less than half the day?”	GAD Hours
9	“Did [you] have multiple worries? Interviewer query: Did [the respondent] exclusively worry about one thing or did [the respondent] have multiple worries?”	Mult Worries
10	“Do you think your worry was excessive; that is, much stronger than it really should be in your situation?”	Excess Worries
11	“How often did you find it difficult to control your worry -- often, sometimes, rarely, or never?”	Control
12	“How often was your worry so strong that you couldn’t put it out of your mind no matter how hard you tried -- often, sometimes, rarely, or never?”	Out of Mind
13	“In the past 12 months, during your period of worry, were you often restless?”	Restless
14	“In the past 12 months, during your period of worry, did you often feel keyed up or on edge?”	Keyed up
15	“In the past 12 months, during your period of worry, did you get tired easily?”	Tired
16	“In the past 12 months, during your period of worry, were you more irritable than usual during this period?”	Irritable
17	“In the past 12 months, during your period of worry, did you often have trouble falling or staying asleep?”	Trouble Sleep
18	“In the past 12 months, during your period of worry, did you often have trouble keeping your mind on what you were doing?”	Keeping Mind
19	“In the past 12 months, during your period of worry, did you often have tense, sore or aching muscles?”	Tense
20	“Think about how your life and activities were affected in the past 12 months by your worry, tension or anxiety. Did these things interfere with your life and activities -- a lot, some, a little, or not at all?”	Interference

*Note that the current question is not in quotes as it is a derivation of two questions: “In the past 12 months, did you have a period when most days you were a lot more worried or tense or anxious than most people would be in your same situation?”, which was responded to positively by all persons who screened into seeing this question.

#### Actigraphy

Participants wore the ActiGraph AM-7164 (formerly the CSA/MTI AM-7164), manufactured by ActiGraph of Ft. Walton Beach, FL. This captures movement intensity emitted in 1-minute intervals. Participants wore them via an elasticized fabric belt which was custom-fitted for each subject and worn on participants’ right hips. Participants were told to keep the device dry (i.e. removing the device before bathing or swimming) and to remove the device before bedtime.

#### Planned analyses

##### Feature engineering

Before performing the analyses, several features were calculated including the wear time of the actigraphy periods and the bouts of wear time [49, 50], entropy, lags [52], stability, seasonal and trends (via seasonal and trend decomposition), nonlinearity (via the Teräsvirta’s nonlinearity test), binary entropy, long-term memory of the time series (via the Hurst coefficient) [53], heterogeneity of the time series (first removing the mean, trend, and autoregressive (AR) information and then computing the generalized autoregressive conditional heteroskedasticity process) [54], number of flat spots (i.e. by dividing the time series into 10 and estimating the longest numbers of consecutive numbers of integers), distributional features of the time series [55, 56], linear trend (via Holtz’s linear trend method) [57], variances of tiled windows, stationarity features [55, 56], nonlinear lagged relationship of a time series [55], mean, standard deviation, root mean square of successive differences, quantiles (10th, 30th, 70th, and 90th quantiles), and spectral power.

##### Machine learning

The current results were analyzed using an ensemble learning model, including two higher-order ensembles that were averaged and 100 lower-order models (each trained on separate machine learning features). In each of the models, the GAD symptom severity was predicted. All model results are based on four-fold cross validation and on out-of-sample predictions. Here the data were split into four random subsets and the data were trained on three-fourths of the data and evaluated on a held-out fourth of the data. The process was then repeated three additional times (one for each fold). All lower-level and higher-order ensembles utilized extreme gradient boosting (“xgboost”) as it tends to outperform other models in most scenarios and has shown to be a robust modeling framework in similar problems. All analyses used risk scores based on the predictions from the ensemble models. Hypothesis 1 was evaluated by calculating the correlation between the predicted and observed GAD symptom severity. Hypothesis 2 was evaluated by normalizing the risk scores to percentiles and then calculating the AUC, sensitivity, and specificity at the optimal cutpoint for elevated GAD symptoms (i.e. 1 standard deviation above the population mean). Hypothesis 3 was evaluated by calculating the odds ratios of having elevated in GAD symptoms at each percentile increase in the risk score. Lastly, Hypothesis 4 was evaluated by comparing the individual correlations of the risk score with each individual GAD symptom.

## Results

### Hypothesis 1: predicting GAD symptom severity across a continuum

**Fig. 1:**
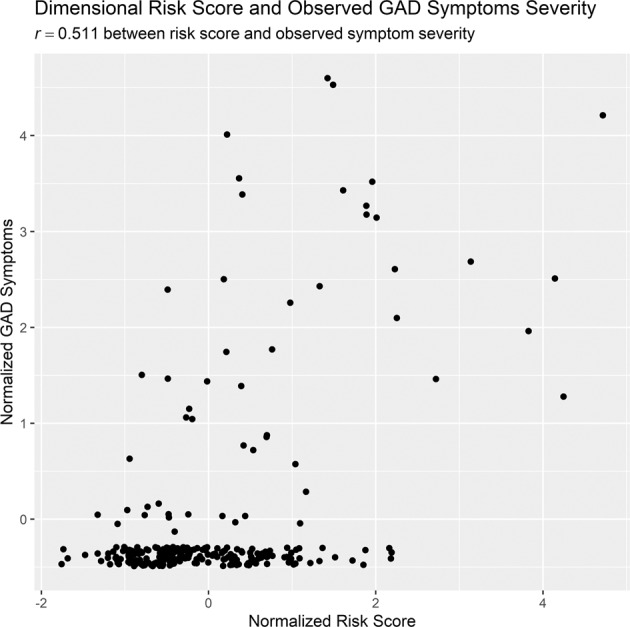
This figure depicts the scatterplot between predicted and observed GAD symptom severity. Note that jitter was added to the current plot so that points were not overlapping.

In predicting the severity of GAD symptoms across a continuum, the calculated risk scores were predictive of GAD severity (*r* = 0.511, *CI* [0.416, 0.595]), in support of Hypothesis 1. See Fig. 1 for the scatterplot between the risk score and the observed GAD symptom severity. The partial correlation was also predictive (*r* = 0.507, *CI* [0.411, 0.592]) even when accounting for age, gender, and ethnicity.

In addition, to examine the discriminant validity of the risk scores, the risk scores were correlated to major depressive disorder symptoms severity and the correlation was much smaller between the predicted GAD risk score and major depressive disorder severity (*r* = 0.190, *CI* [0.071, 0.304]), suggesting that there was strong discriminant validity between predicted risk and GAD symptom severity compared to major depressive disorder symptom severity.

### Hypothesis 2: precision of elevated risk classifications

**Fig. 2:**
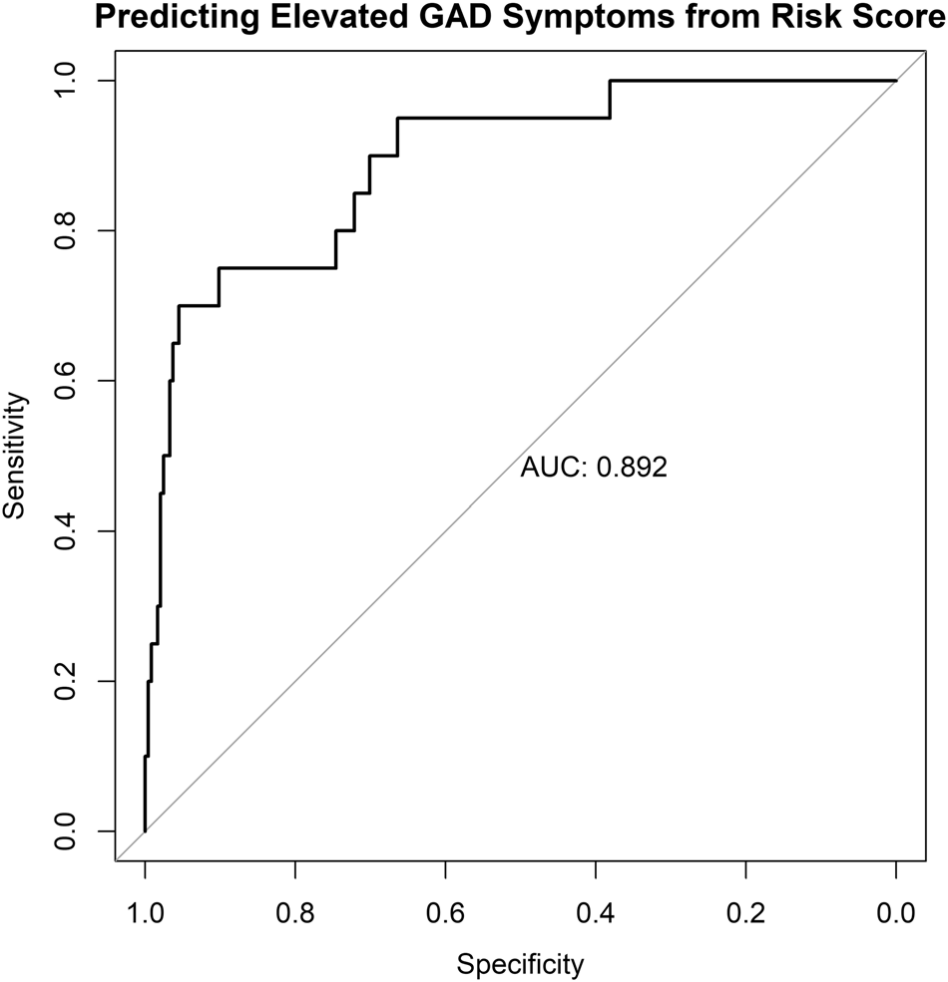
The current plot depicts the receiver operating characteristic curve. The black line describes the differentiating sensitivity and specificity of the risk of elevated symptoms (defined as greater than 1 standard deviation above the population mean in GAD symptoms) across varying levels of the risk scores. Note that the grey line is what would be expected by chance.

Supporting Hypothesis 2, there was a high precision in differentiating those who have elevated symptoms compared to the rest of the population (greater than 1 standard deviation above the population mean in the GAD symptom severity) with an AUC of 0.892, 70.0% sensitivity, 95.5% specificity, and a Brier Score = 0.092). See Fig. 2 for a plot of the receiver operator curve.

### Hypothesis 3: high risk scores predict high odds of elevated GAD symptoms

**Fig. 3:**
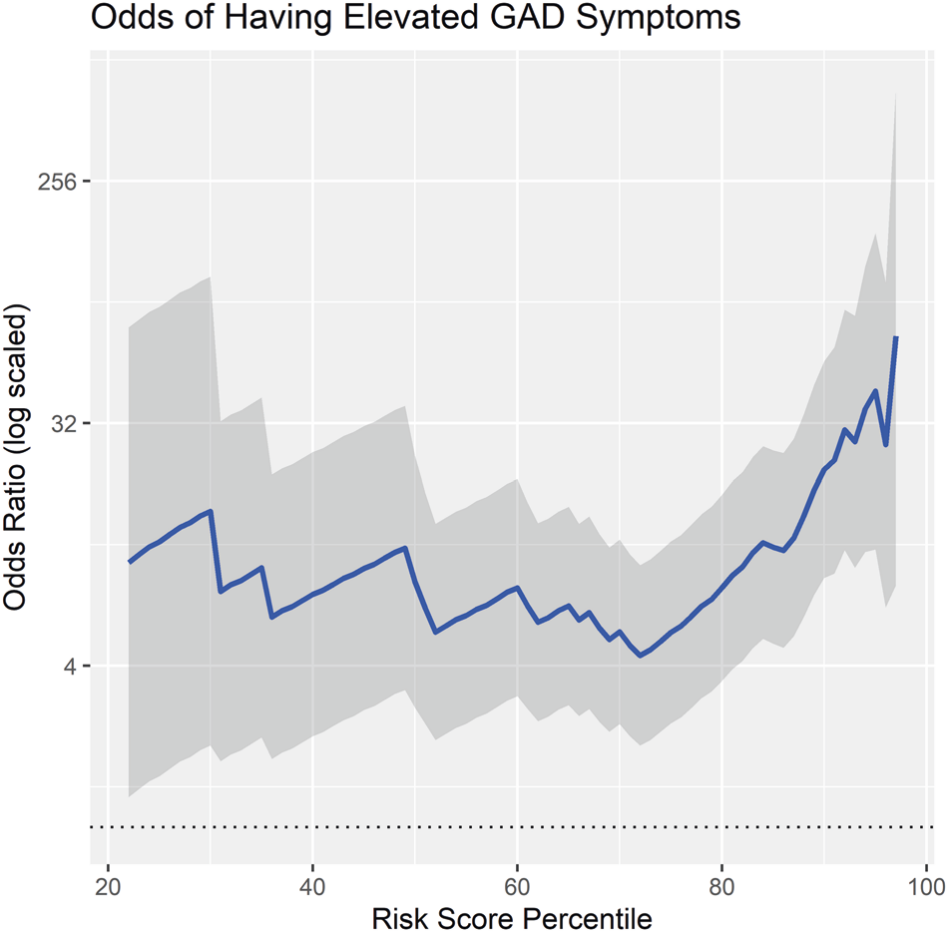
This plot depicts the odds of having elevated GAD symptoms (defined as greater than 1 standard deviation above the population mean in GAD symptoms) at varying levels of risk scores. The gray lines depict the 95% confidence intervals of the odds ratios.

**Fig. 4:**
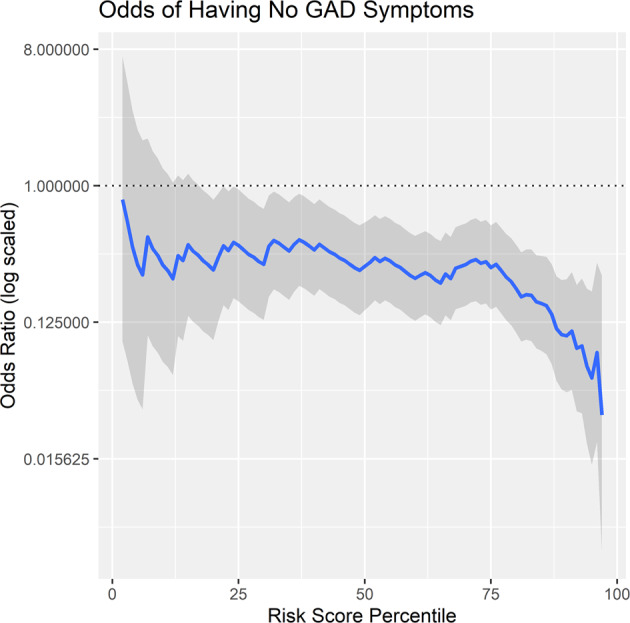
This plot depicts the odds of reporting no GAD symptoms (defined as declining all symptom criteria) at varying levels of risk scores. The gray lines depict the 95% confidence intervals of the odds ratios.

At the 90th or greater percentile, the odds of having elevated GAD was 21.527 *CI* [8.504, 54.497], which supports Hypothesis 3. See Fig. 3 for a plot of the continuous odds ratio plot of elevated risk. compared to all lower scores. Notably, the inverse was also calculated (i.e. predicting the odds of having no GAD symptoms across the continuum), and the odds of having no reported symptoms when at the 90th or greater percentile on the risk score was 0.102 CI [0.043, 0.241]. See Fig. 4. This suggests that those at high risk scores at a 21-fold increased risk of having elevated GAD symptoms and 90% less likely to have no GAD symptoms.

### Hypothesis 4: risk scores predicting individual GAD symptoms

**Fig. 5:**
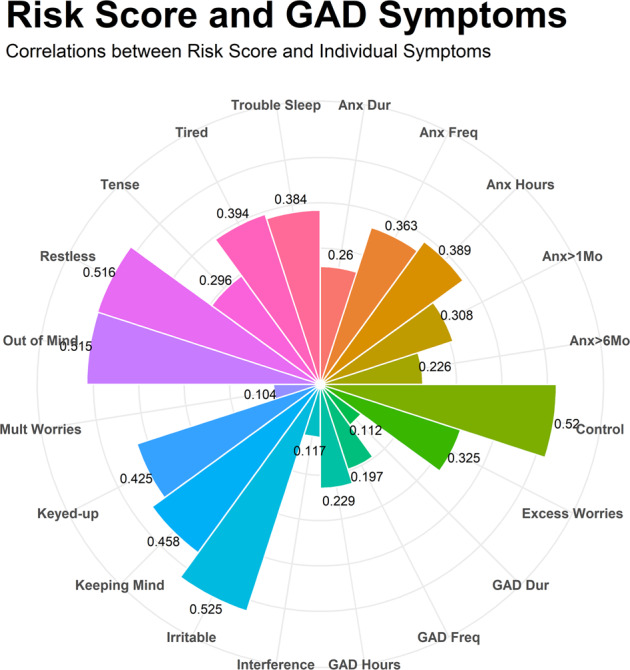
Correlations between the Risk Score and Individual GAD Symptoms. This plot depicts the strength of correlations between the risk score and individual GAD symptoms.

Interestingly, only partially supporting Hypothesis 4 (i.e. that risk scores would more strongly relate to somatic symptoms, compared to more cognitive symptoms), risk scores were most strongly correlated with irritability (*r* = 0.525), controllability (*r* = 0.520), restlessness (*r* = 0.516), difficulty putting worries out of mind (*r* = 0.515), keeping mind focused (*r* = 0.458), keyed up (*r* = 0.425), tired (*r* = 0.394), hours per day with anxiety (*r* = 0.389), trouble sleeping (*r* = 0.384), anxiety frequency (*r* = 0.363), excessive worries (*r* = 0.325), and being anxious for more than 1 month (*r* = 0.308). The only GAD symptoms which were not significantly correlated with the risk scores were multiple worries (*r* = 0.104), duration of having uncontrollable worries more than most (*r* = 0.112), and interference (*r* = 0.117). Of note, these associations could be affected by a potential range restriction among some symptoms (see supplementary Table 1 for means, standard deviations, and correlations of the symptoms). See Fig. 5 for a plot of all symptom correlations. Thus, partially supporting Hypothesis 4, although some somatic symptoms were more strongly related to risk scores than some cognitive symptoms (e.g. restlessness compared to having multiple worries), some cognitive symptoms were more strongly related to risk scores than somatic symptoms (e.g. controllability compared to trouble sleeping).

## Discussion

The current results suggest that machine learning models using movement time series data collected across one week can accurately predict GAD symptom severity across a continuum (*r* = 0.511). In terms of their clinical utility, risk scores from these models accurately detected those with elevated GAD symptoms (AUC = 0.892, 70.0% Sensitivity, 95.5% Specificity, Brier Score = 0.092). Importantly, the current findings complement prior findings that physical activity is related to lower odds of having GAD [58, 59]. In particular, those with a movement risk score at or above the 90^th^ percentile had 21 times the odds of having elevated GAD symptoms compared to those with lower risk scores. The risk scores also showed strong discriminant validity with a weak relationship to major depressive disorder symptoms (*r* = 0.190). In predicting GAD symptoms, the current research also corroborates potential prognostic predictive performance of movement patterns, which could be capturing low intensity movements (e.g., psychomotor agitation) and high intensity movements (e.g., exercise).

These results suggest that movement calculations may have great clinical utility. Given the poor diagnosis rates [60] and care received by persons with GAD, the current research suggests that integrating passive sensing may present the opportunity for non-invasive assessment of GAD symptoms. Moreover, the current findings build upon and extend prior studies which have shown an ability to utilize passively collected movement data to predict major depressive disorder symptom severity, pain, worry severity, and social anxiety [35, 61, 62]. Taken together, the current study may suggest that adding actigraphy risk models to assess psychiatric symptoms could be to increase accurate detection of common mental health disorders and thereby potentially enable patients to seek treatment more quickly. Notably, persons with GAD may be more comfortable than most persons in allowing clinicians to view their sensor data, which may suggest that integrating passive sensor data into care settings could be both acceptable within this patient population [63].

In the current study, the findings also highlighted the importance of studying individual GAD symptoms in addition to total symptom severity [41–43]. Similar to prior studies showing differential risk and consequence of individual GAD symptoms [44, 45], the present study found that the risk scores held different relationships to GAD symptoms. The strongest relationships between individual GAD symptoms and the risk scores were irritability, restlessness, and the controllability of worry. Corroborating the link between risk scores derived from movement data, prior studies have shown a strong link between anger and physiological arousal following exercise [64], and experimental evidence suggests that anger may actually be reduced among persons in an exercise condition [65]. Interestingly, restlessness in the extreme form (i.e. restless leg syndrome) has also been shown to be affected by exercising [66]. Thus, the current findings further corroborate prior findings suggesting that movement patterns may be tied to important GAD symptoms.

Although there was clear clinical utility of these risk models, the risk scores did not significantly relate to all individual GAD symptoms, including experiencing multiple worries, duration of uncontrollable worries, and lifetime interference. Nevertheless, the current models did relate to other assessments regarding the duration of worry and anxiety. Moreover, although impairment is one of the criteria for GAD symptoms, this symptom is not absolutely required to be diagnose GAD (as GAD can also be diagnosed upon experiencing clinically significant distress even in the absence of clinical impairment) [4]. However, the lack of an association between the risk score and the presence of multiple worries is an important limitation in the current models, as the presence of multiple worries is required for a current diagnosis of GAD. Nevertheless, at least in network models, the experience of multiple worries appears to be the least central of the GAD symptoms [67]. Thus, these findings suggest that these risk scores should not be used as in isolation to assess all GAD criteria and infer diagnoses based on the current formulation of GAD. In the space of passive sensing, future studies should examine whether some of these symptoms may be better assessed through the addition of other sensor types (e.g. monitoring social interactions may provide insight into the interference of these worries onto one’s life).

The current study has several notable strengths. Firstly, GAD symptoms were assessed via a gold-standard semi-structured clinical interview. Secondly, the current sample included a large and diverse national cohort. Moreover, the current approach collected prospective data within the context of persons’ daily lives. Lastly, the current results utilized cutting-edge machine learning techniques with out-of-sample cross-validated predictions to minimize the possibility of overfitting.

### Limitations

Despite the present study’s several strengths, it also has limitations; in particular, the current study examined the detection of GAD symptom severity rather than prognostically predicting the course of GAD symptom severity across time. Moreover, the instant sample of data did not incorporate nighttime actigraphy (as participants were asked not to wear the actigraphs at night). In addition, the current study did not examine discriminant validity among anxiety disorders, and as such future work should examine whether other anxiety disorders can be differentiated from GAD using actigraphy data. Of note, a sizeable portion of the sample did not wear the actigraphs for a sufficient amount of time to be included in the modeling, and consequently future work should examine everyday devices that are further integrated into persons’ daily lives (i.e. smartphones). Note that some of the findings linking the risk score to particular symptoms of GAD may have been impacted by a range restriction. For instance, controllability had a higher relationship to the risk score than trouble sleeping, and controllability had a higher variance than trouble sleeping (see supplementary Table 1).

## Conclusions

Taken together, this study suggests that there is important clinical utility in utilizing movement in daily life accompanied with machine learning models to predict the severity of GAD symptoms. Given this, future studies should examine the potential additive predictive value of continuous movement monitoring which could assess both daytime and nighttime movement patterns in predicting GAD symptom severity. Future studies should build upon the current study and continue to examine the utility of movement and other types of passive sensor data to predict both the diagnosis, symptom severity, and course of GAD symptoms across time.

## Supplementary information

Means, standard deviations, and correlations among GAD Items

## Data Availability

All data are publicly available through Inter-university Consortium for Political and Social Research.

## Code availability

The code for the present manuscript is not publicly available.
