# Supplementary material for: Digital phenotyping of generalized anxiety disorder: using artificial intelligence to accurately predict symptom severity using wearable sensors in daily life
Source: Transl Psychiatry. 2022 Aug 17;12:336. doi: 10.1038/s41398-022-02038-1 (PMC9385727; doi:10.1038/s41398-022-02038-1)
Supplement: Supplementary file 1 — Means, standard deviations, and correlations among GAD Items [file 41398_2022_2038_MOESM1_ESM.pdf]

Supplemental Table 1

Means, standard deviations, and correlations among GAD Items

Variable	*M*	*SD*	1	2	3	4	5	6	7	8	9	10	11	12	13	14	15	16	17	18	19
1. Anx>1Mo	0.14	0.35																			
2. Anx>6Mo	0.03	0.17	.43																		
3. Anx Dur	0.52	1.81	.70	.74																	
4. Anx Freq	0.38	1.04	.90	.51	.73																
5. Anx Hours	0.24	0.73	.80	.55	.74	.89															
6. GAD Dur	0.11	0.58	-.08	-.03	-.05	-.07	-.06														
7. GAD Freq	0.12	0.59	-.09	-.04	-.06	-.08	-.07	.80													
8. GAD Hours	0.08	0.47	-.07	-.03	-.05	-.06	-.05	.75	.92												
9. Mult Worries	0.05	0.21	.38	.39	.30	.38	.33	.28	.30	.23											
10. Excess Worries	0.03	0.16	.34	.11	.29	.33	.37	.01	.05	.07	-.04										
11. Control	0.25	0.84	.56	.24	.45	.63	.70	.17	.22	.30	-.06	.57									
12. Out of Mind	0.23	0.78	.59	.26	.49	.65	.73	.09	.17	.23	-.06	.59	.96								
13. Restless	0.07	0.26	.51	.21	.42	.56	.61	.15	.24	.32	-.06	.59	.88	.86							
14. Keyed-up	0.06	0.24	.53	.23	.49	.61	.64	.01	.08	.12	-.06	.45	.79	.82	.73						
15. Tired	0.06	0.23	.51	.24	.43	.59	.64	.13	.15	.20	-.05	.27	.78	.77	.75	.49					
16. Irritable	0.08	0.27	.54	.20	.45	.58	.65	.15	.23	.31	-.06	.58	.94	.93	.92	.83	.73				
17. Trouble Sleep	0.05	0.21	.48	.28	.48	.50	.65	-.01	.08	.08	-.05	.30	.74	.75	.64	.71	.65	.69			
18. Keeping Mind	0.08	0.27	.54	.20	.45	.58	.67	.15	.23	.31	-.06	.49	.90	.93	.86	.83	.73	.95	.69		
19. Tense	0.04	0.19	.37	.20	.45	.42	.53	.17	.20	.26	-.04	.21	.67	.68	.56	.53	.72	.62	.72	.69	
20. Interference	0.01	0.11	.16	-.02	.21	.10	.21	.29	.16	.28	-.02	.20	.35	.29	.38	.27	.28	.37	.32	.37	.35

Note. *M* and *SD* are used to represent mean and standard deviation, respectively.
